# Supplementary figures and images for: RNF213 regulates blood‒brain barrier integrity by targeting TRAF3 for type I interferon activation during A. baumannii infection
Source: PLoS Pathog. 2025 Jul 7;21(7):e1013333. doi: 10.1371/journal.ppat.1013333 (PMC12251097; doi:10.1371/journal.ppat.1013333)

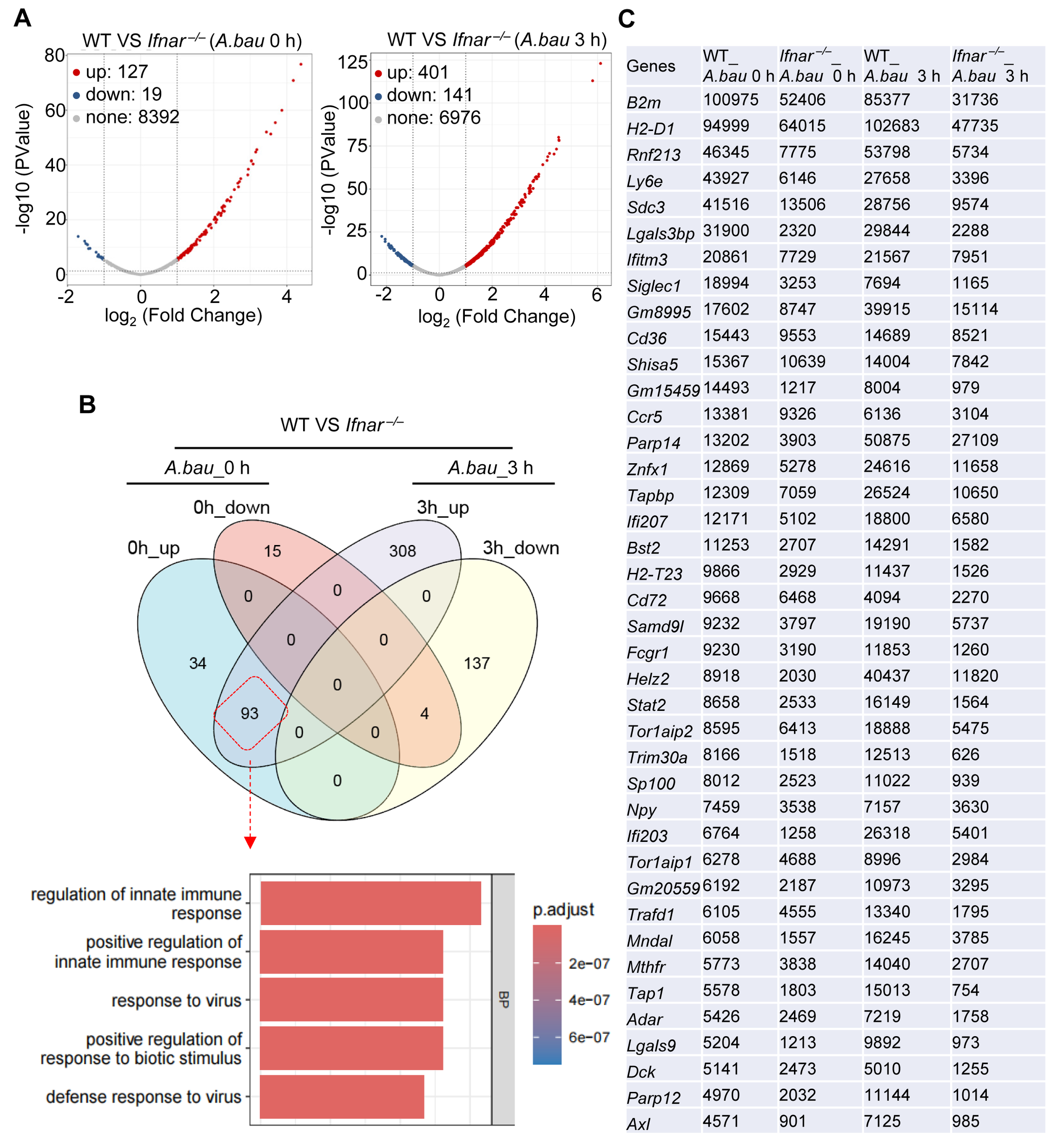

Supplement: S1 Fig — (A) RNA sequencing analysis of gene expression in uninfected and A. baumannii infected WT and Ifnar–/– BMDMs for 3 hours. Differentially expressed genes in WT and Ifnar–/– BMDMs are shown. The volcano plots display the distribution of DEGs in WT and Ifnar–/– samples at 0 h (left) and 3 h (right) post infection. The dotted vertical lines indicate the fold-change threshold (±1 log₂FC), while the horizontal dotted line represents the significance threshold (P = 0.05). (B) Enrichment analysis of the signaling pathways that were highly expressed in WT BMDMs compared with Ifnar–/– BMDMs. (C) The top 40 genes ranked differentially expressed genes in WT versus Ifnar–/– BMDMs under both uninfected and A. baumannii-infected conditions, with ranking determined by basal expression in uninfected WT cells. (TIF) [file ppat.1013333.s001.tif]

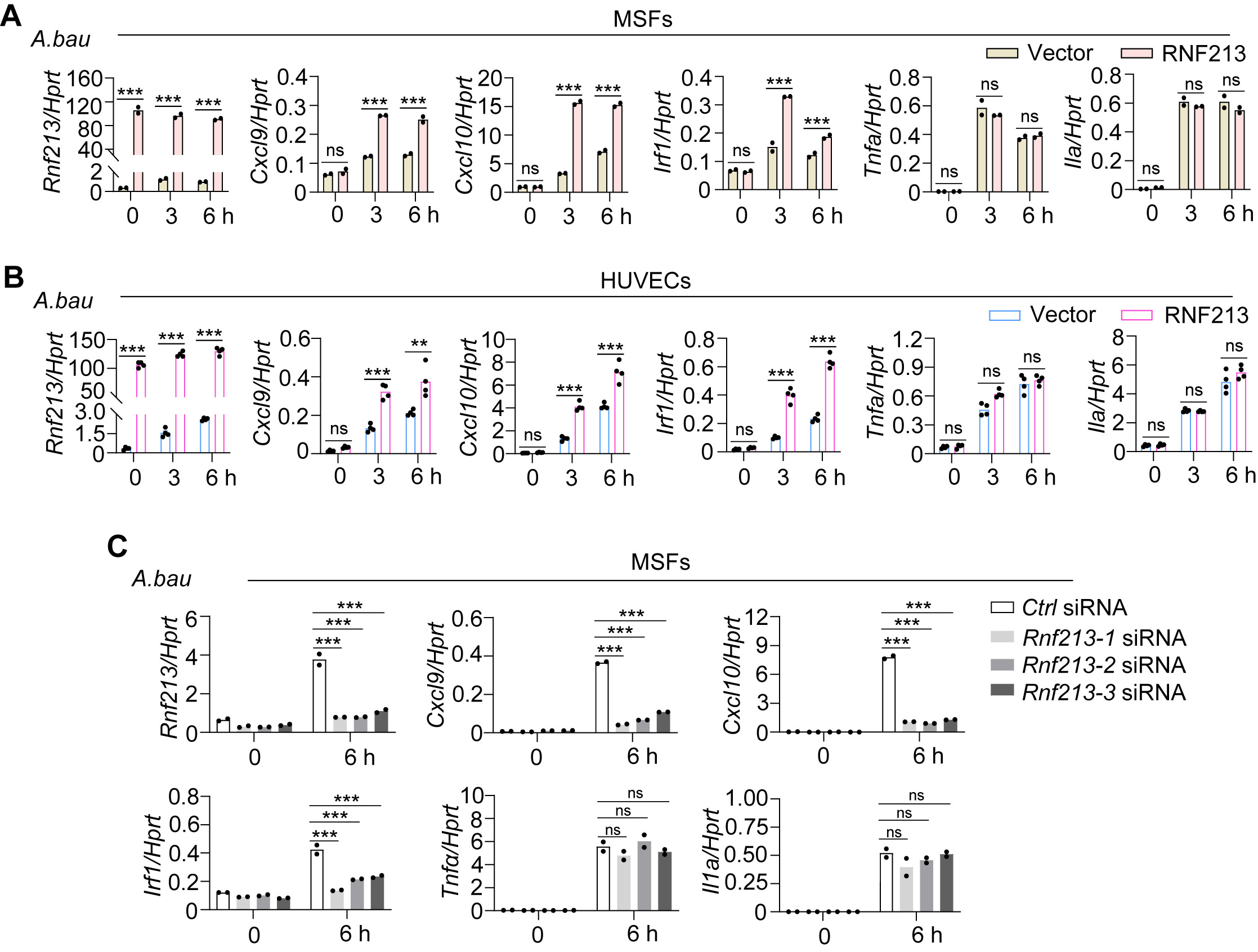

Supplement: S2 Fig — (A) qRT-PCR analysis of Rnf213, Cxcl9, Cxcl10, Irf1, Tnfa, and Il1a in WT MSFs transfected with RNF213 or Vector control, without treatment or infected with A. baumannii (50 MOI) for indicated times (n = 2 technical replicates; 3 independent experiments). (B) qRT-PCR analysis of Rnf213, Cxcl9, Cxcl10, Irf1, Tnfa, and Il1a in HUVECs transfected with RNF213 or Vector control, without treatment or infected with A. baumannii (50 MOI) for indicated times (n = 4 technical replicates; 3 independent experiments). (C) qRT-PCR analysis of Rnf213, Cxcl9, Cxcl10, Irf1, Tnfa, and Il1a in WT MSFs transfected with control siRNA or siRNAs specific to Rnf213 without treatment or infected with A. baumannii (50 MOI) for indicated times (n = 2 technical replicates; 3 independent experiments). Data are representative of 3 independent experiments with similar results (A-C). Data represent Mean ± SEM for (A-C), 2-sided Student’s t-test without multiple-comparisons correction, **P < 0.01; ***P < 0.001; ns, not significant. (TIF) [file ppat.1013333.s002.tif]

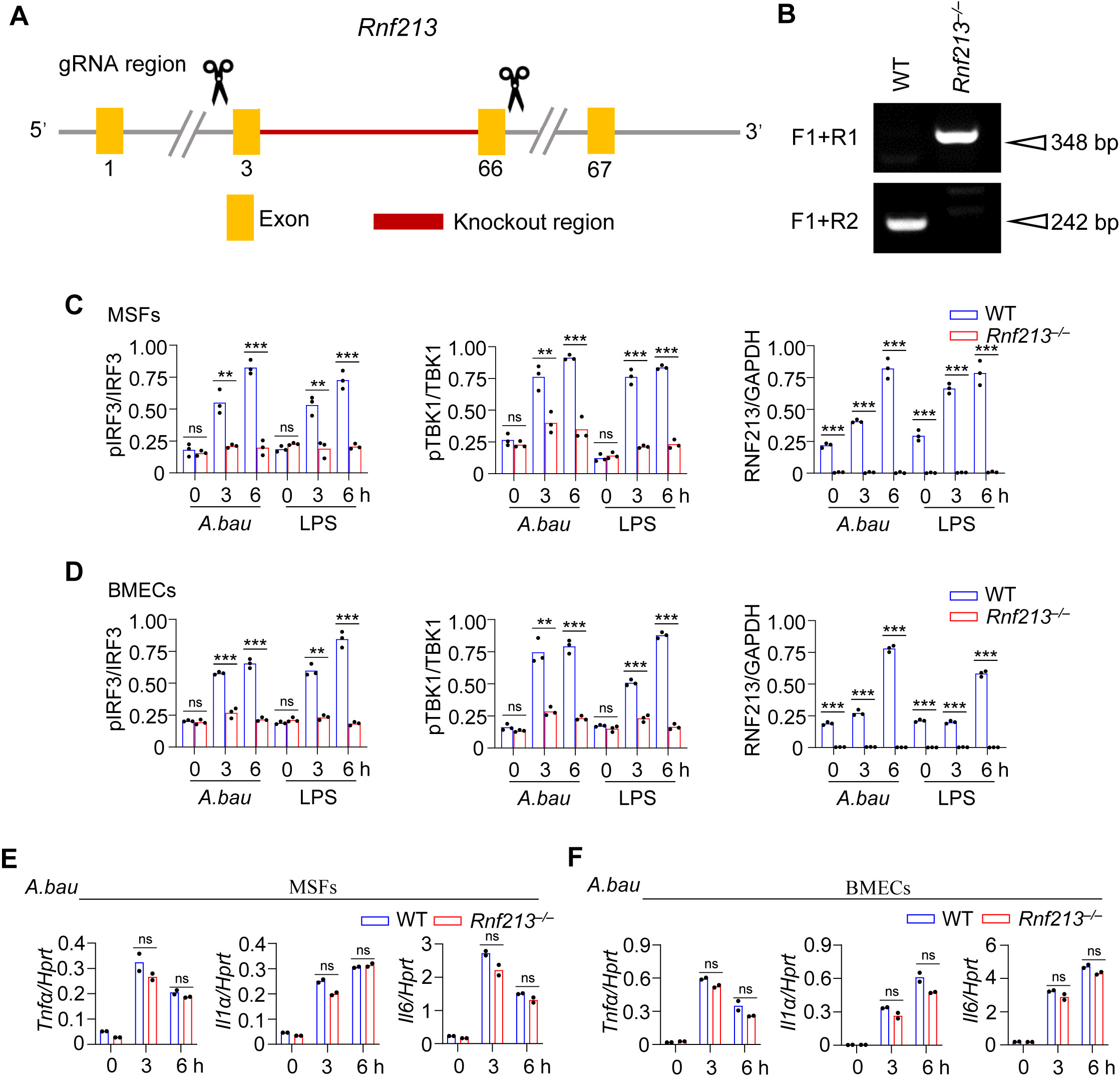

Supplement: S3 Fig — (A-B) Targeting strategy used to generate Rnf213–/– mice (A) and genotyping of offspring generated from breeding of Rnf213 heterozygous mice (B). (C-D) Quantification analysis of phosphorylation of IRF3 and TBK1, and total IRF3, TBK1, and RNF213 in WT and Rnf213–/– MSFs (C) and BMECs (D) without treatment or infected with A. baumannii (50 MOI), or stimulated with LPS (200 ng/mL) for indicated times (n = 3 independent experiments). (E-F) qRT-PCR analysis of Tnfa, Il1a, and Il6 in WT and Rnf213–/– MSFs (E) and BMECs (F) without treatment or infected with A. baumannii (50 MOI) as indicated times (n = 2 technical replicates; 3 independent experiments). Data are from 3 independent experiments (C, D) or representative of 3 independent experiments with similar results (B, E-F). Data represent Mean ± SEM for (C-F), 2-sided Student’s t-test without multiple-comparisons correction, **P < 0.01; ***P < 0.001; ns, not significant. (TIF) [file ppat.1013333.s003.tif]

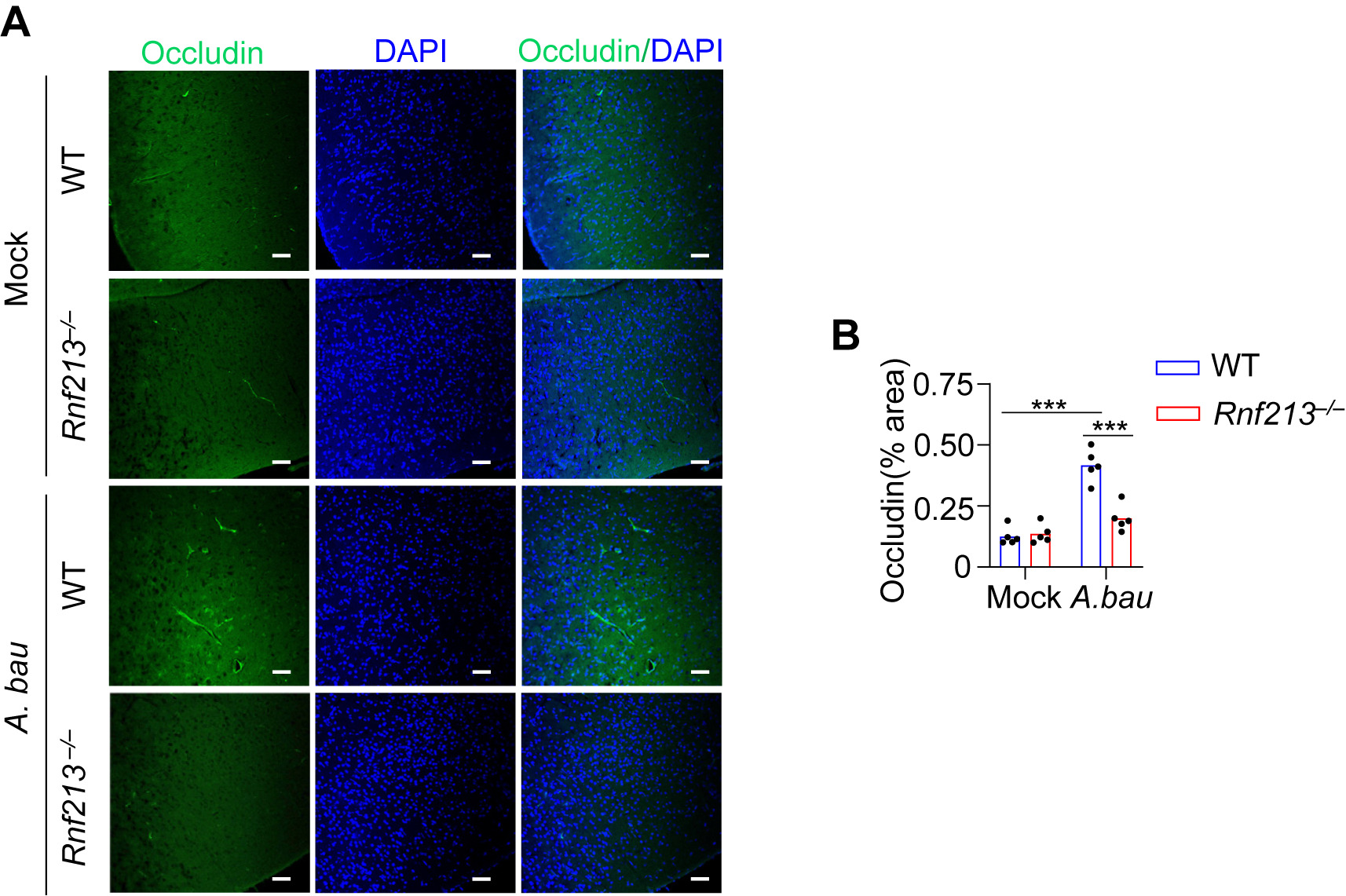

Supplement: S4 Fig — (A-B) Representative confocal microscopy images (A) and quantification analysis (B) showing immunostaining for Occludin in the brain of uninfected and A. baumannii-infected mice in Fig 3B. Data are representative of 3 independent experiments with similar results (A-B). Each dot represents one field and total 5 random fields were quantified for (B). Scale bar: 50 μm for (A). Data represent Mean ± SEM for (B), 2-sided Student’s t-test without multiple-comparisons correction, ***P < 0.001. (TIF) [file ppat.1013333.s004.tif]

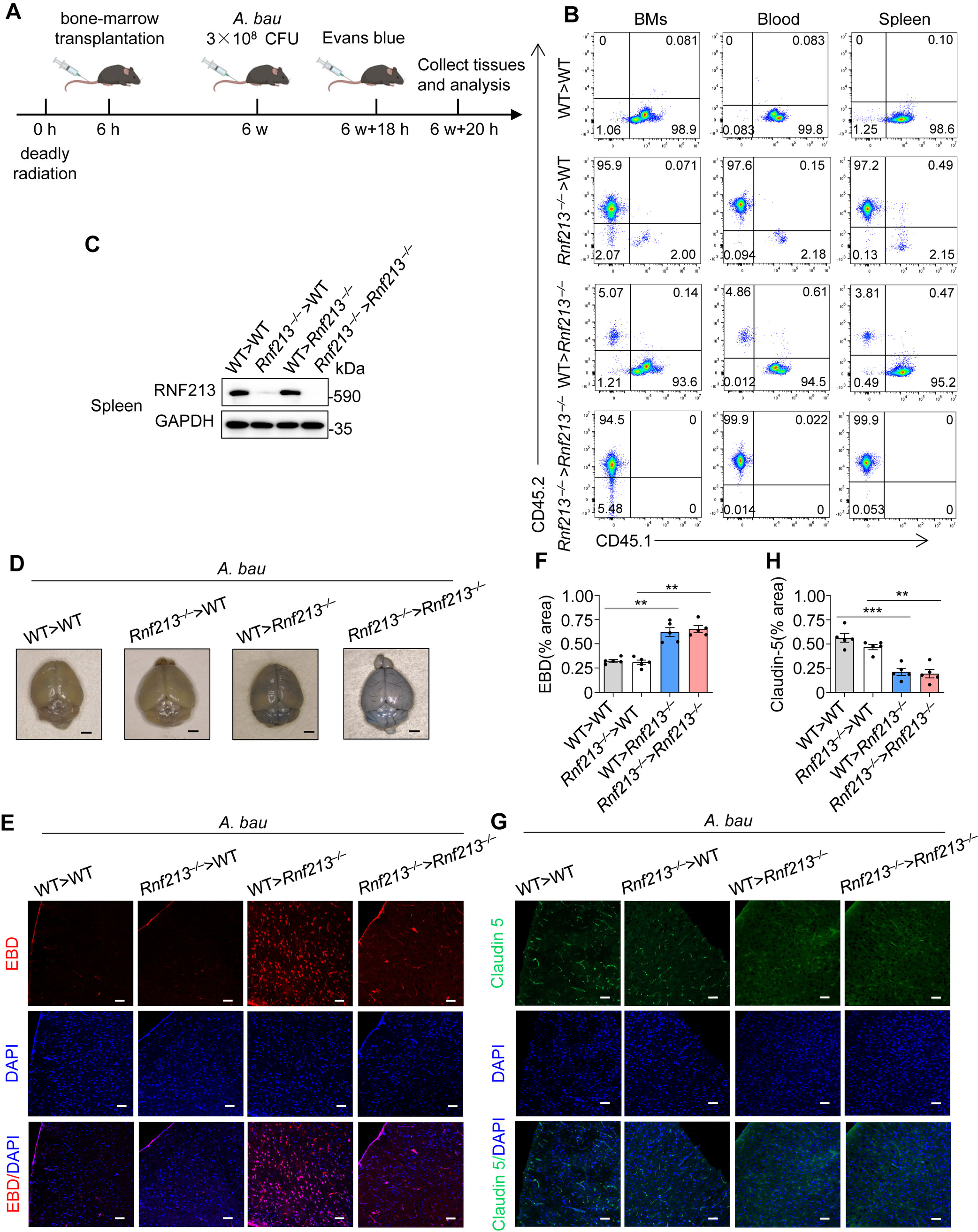

Supplement: S5 Fig — (A) Strategy for the procedure of bone marrow transplantation and A. baumannii infection experiments. Six to eight hours prior to bone marrow transplantation, the recipient mice were irradiated with a dosage of 10 Gy for 10 minutes. For transplantation, 10 million bone marrow cells were injected into the tail veins of the recipient mice. Six weeks later, bone marrow chimeric mice were intravenously infected with A. baumannii and colony-forming units (CFUs) were analyzed at 20 hours after infection. Evans blue dye (EBD) was intravenously injected into mice at 18 hours after infection and leakage into the brain was evaluated 2 hours later. (B) Representative flow cytometry plots of CD45.1+ and CD45.2+ cells in bone marrow, peripheral blood and spleens from chimeric mice as indicated. (C) Immunoblot analysis of RNF213 in the spleen of chimeric mice. (D-F) Representative microscopy images (D) and confocal microscopy images (E), and quantification analysis of EBD (F) showing vessel leakage in the brain of uninfected and A. baumannii-infected mice assessed by EBD. (G-H) Representative confocal microscopy images (G) and quantification analysis (H) showing immunostaining for Claudin-5 in the brain of uninfected and A. baumannii-infected mice. Data are representative of 3 independent experiments with similar results (B-H). Each dot represents one field and total 5 random fields were quantified for (F) and (H). Scale bar: 20 mm for (D), 50 μm for (E, G). Data represent Mean ± SEM for (F, H), 2-sided Student’s t-test without multiple-comparisons correction, **P < 0.01; ***P < 0.001. A was created with Biorender.com. (TIF) [file ppat.1013333.s005.tif]

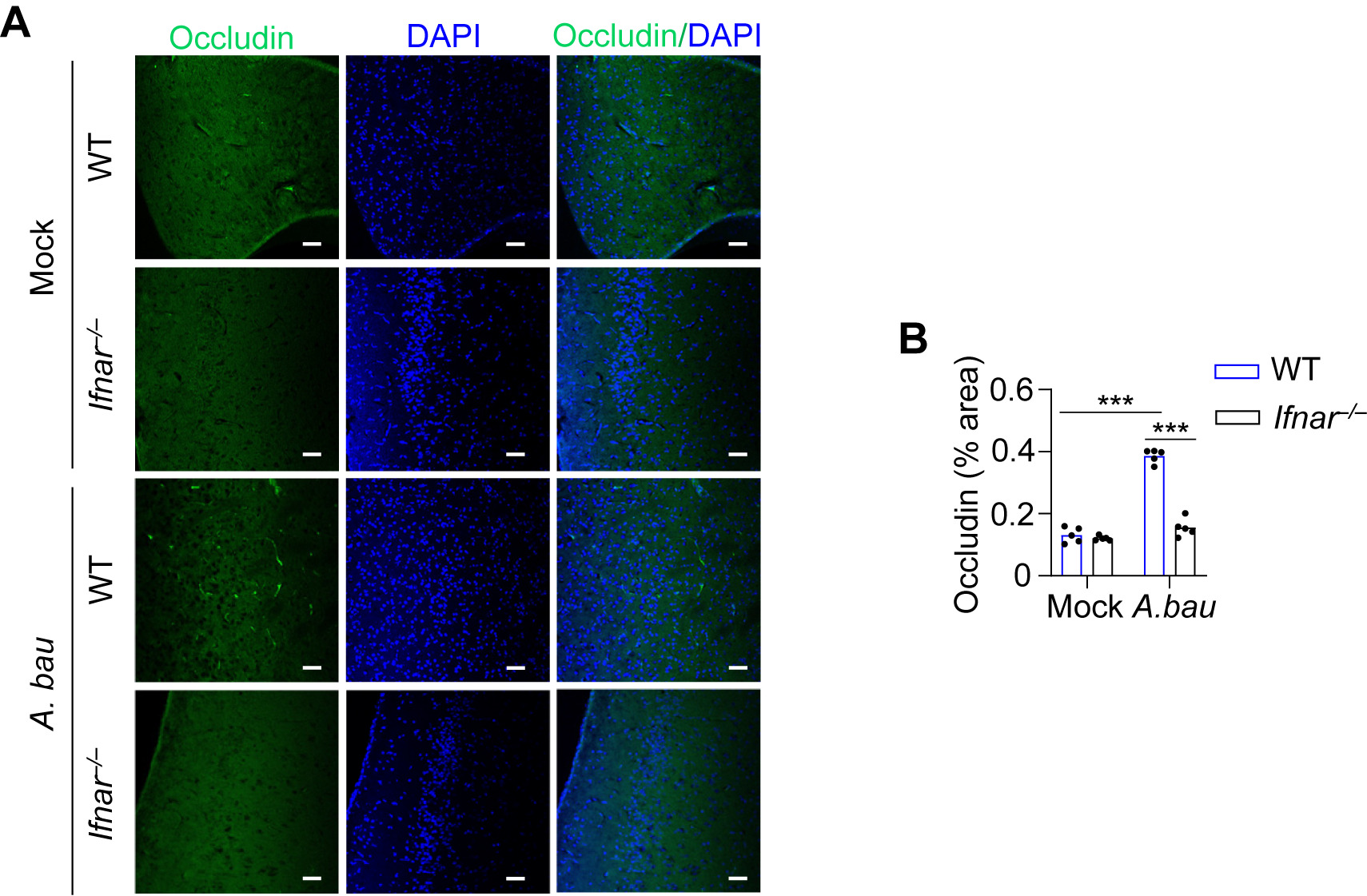

Supplement: S6 Fig — (A-B) Representative confocal microscopy images (A) and quantification analysis (B) showing immunostaining for Occludin in the brain of uninfected and A. baumannii-infected mice in Fig 4D. Data are representative of 3 independent experiments with similar results (A-B). Each dot represents one field and total 5 random fields were quantified for (B). Scale bar: 50 μm for (A). Data represent Mean ± SEM for (B), 2-sided Student’s t-test without multiple-comparisons correction, ***P < 0.001. (TIF) [file ppat.1013333.s006.tif]

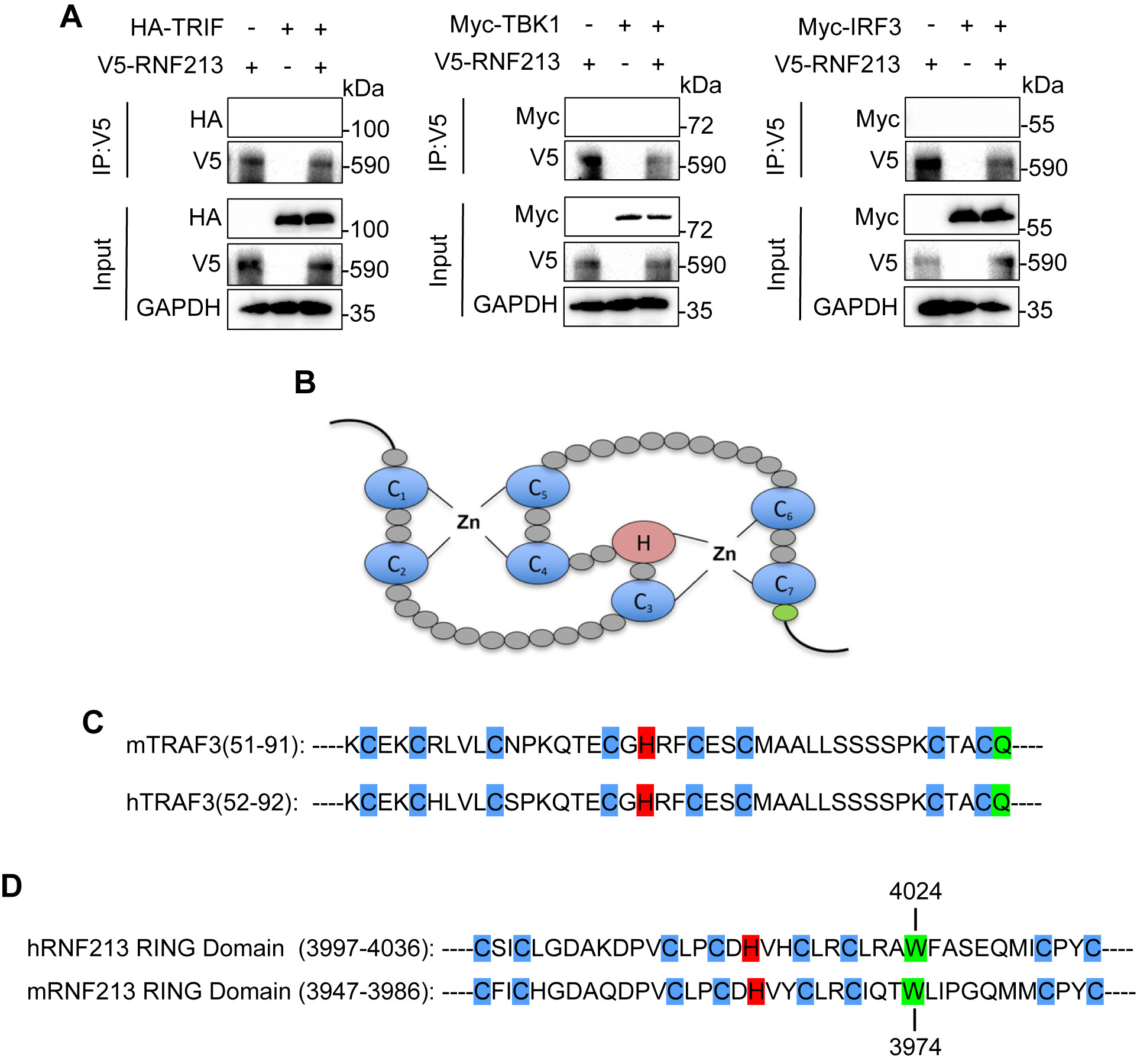

Supplement: S7 Fig — (A) Immunoblot analysis of V5-RNF213 co-IP with HA-TRIF, Myc-TBK1 and Myc-IRF3 from lysates of HEK293T cells transfected with plasmids as indicated. (B) Representation of the ‘cross-brace’ structure of the C3HC4 RING-finger domain, mediated through cysteine and histidine Zinc-binding. (C) Sequence alignment of mouse TRAF3 (mTRAF3, 51–91) and human TRAF3 (hTRAF3, 52–92) domains. (D) Sequence alignment of human RNF213 (hRNF213) RING and mouse RNF213 (mRNF213) RING domains. Data are representative of 3 independent experiments with similar results(A). (TIF) [file ppat.1013333.s007.tif]

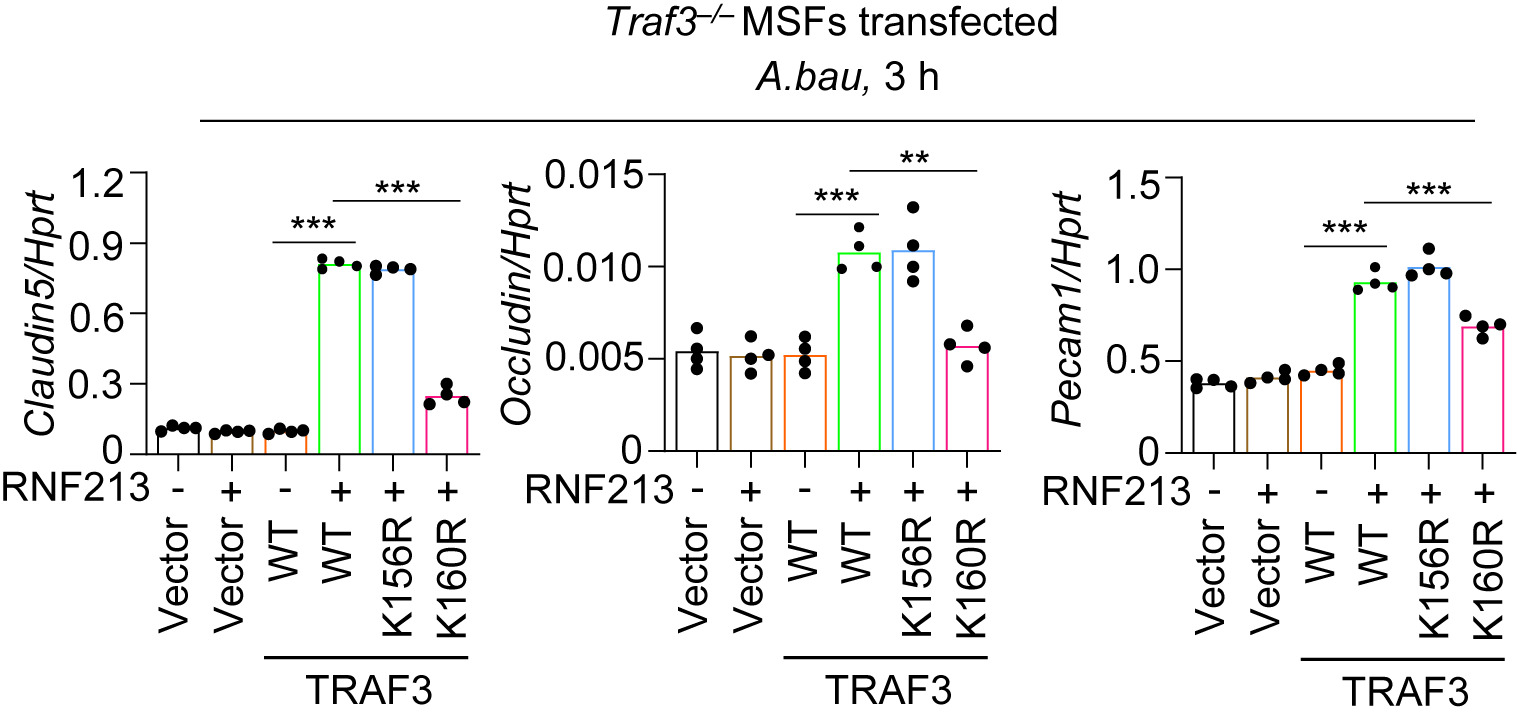

Supplement: S8 Fig — qRT-PCR analysis of Claudin-5, Occludin, and Pecam1 in Traf3–/– MSFs transfected with RNF213 combined with WT or mutant TRAF3 plasmids (K156R and K160R), infected with A. baumannii (50 MOI) for indicated times. (n = 4 technical replicates; 3 independent experiments). Data are representative of 3 independent experiments with similar results. Data represent Mean ± SEM, 2-sided Student’s t-test without multiple-comparisons correction, **P < 0.01; ***P < 0.001. (TIF) [file ppat.1013333.s008.tif]
