# Supplementary material for: RNF213 regulates blood‒brain barrier integrity by targeting TRAF3 for type I interferon activation during A. baumannii infection
Source: PLoS Pathog. 2025 Jul 7;21(7):e1013333. doi: 10.1371/journal.ppat.1013333 (PMC12251097; doi:10.1371/journal.ppat.1013333)
Supplement: Attachment — Submitted filename: RNF213 Response to Reviewers_250416.doc [file ppat.1013333.s011.doc]

Thank you for submitting your manuscript to PLOS Pathogens. Your manuscript has now been seen by three reviewers, who recognized the conceptual importance of your work and who commented positively on certain aspects of your manuscript while criticizing others. After careful consideration, we feel that your manuscript has merit but does not fully meet PLOS Pathogens's publication criteria as it currently stands. Therefore, we invite you to submit a revised version of the manuscript that addresses the points raised during the review process. Of particular importance are the missing transplantation controls highlighted by Reviewer 1 and 3 and experiments on the mechanism and consequences of ubiquitylation as suggested by all three reviewers.

1. **We performed bone marrow transplantation (BMT) between WT (CD45.1^+^) and *Rnf213^–/–^* (CD45.2^+^) mice to examine the engraftment efficiency. Six weeks after transplantation, we performed flow cytometry analysis and confirmed that donor-derived bone marrow cells were predominantly existed in the bone marrow, blood, and spleen of recipient mice. In addition, we performed western blot analysis of RNF213 in chimeric mice, and found that RNF213 was notably detected in the spleen from *Rnf213^–/–^* mice that received bone marrow from WT mice, but not detected in the spleen from WT mice that received bone marrow from *Rnf213^–/–^* mice. These data collectively indicate that the transplantation system is successful in our experiments.**
2. **We have designed additional experiments to address this question. We transfected WT and mutant TRAF3 (K156R, K160R) into *Traf3^–/–^* MSFs and evaluated ISGs and TJ-related proteins expression in response to RNF213 overexpression. We found that the expression of *Ifnb, Cxcl9, Cxcl10, Claudin-5, Occludin* and *Pecam1* was significantly higher in *Traf3^–/–^* MSFs co-transfected with WT TRAF3 or K156R mutant of TRAF3 and RNF213 than those in *Traf3^–/–^* MSFs co-transfected with K160R mutant of TRAF3 and RNF213 in response to *A. baumannii* infection. Consistently, the K160R mutation of TRAF3 impaired its ability to activate TBK1 and IRF3 compared to WT TRAF3 and K156R.**

**Please check our responses to all the specific aspects below:**

Reviewer #1:

**Summary**

Li et al. report that RNF213, previously known for its association with moyamoya disease, plays an important role in protecting the blood-brain barrier during Acinetobacter baumannii infection. Using a myriad of experimental approaches, they demonstrate that interferon signaling induces RNF213 expression, which then interacts with TRAF3 to mediate K27-linked polyubiquitination at K160. This molecular interaction promotes type I interferon signaling and regulates tight junction proteins including Claudin-5, Occludin, and Pecam1. They additionally demonstrate that the protective effects of RNF213 are specific to non-myeloid cells, where loss leads to compromised blood-brain barrier integrity and exacerbated A. baumannii infection. This study establishes a mechanistic link between RNF213 and blood-brain barrier function through TRAF3-mediated signaling, providing insights into both moyamoya disease pathogenesis and potential therapeutic strategies for CNS bacterial infections.

**We thank the reviewer found our study important. We have revised our manuscript and added alternative explanations in our manuscript.**

**Major Issues: Key Experiments Required for Acceptance**

1. The authors claim RNF213 expression is “dependent” on IFNAR signaling (lines 37-38), but the data show reduced but not absent expression in IFNAR knockout cells. The terminology should be modified to “partially dependent” or “significantly regulated by” to more accurately reflect the data.

**We thank the reviewer for this suggestion and have changed the sentence to “increased expression of *Rnf213* is significantly regulated by interferon alpha/beta receptor (IFNAR) signaling” in the abstract section and made changes accordingly throughout the manuscript.**

2. The mechanism for RNF213 in directly ubiquitinating targets in IFN-I signaling is unclear. Including ubiquitination experiments to identify direct substrates of RNF213 or further explore pathways altered by RNF213 deficiency in the RNA-seq, may be helpful in discerning this mechanism.

**We sincerely thank the reviewer for your insightful comments regarding the mechanism for RNF213 in directly ubiquitinating targets in IFN-I signaling. We agree with you that investigations by using RNA-seq and more ubiquitination screening experiments will be helpful to further confirm the critical role of RNF213 in IFN-I signaling pathway through ubiquitination activity.**

**In this manuscript, our data revealed RNF213 deficiency caused impaired ISGs expression in response to *A. bau* infection and LPS stimulation by using qRT-PCR. Furthermore, we confirmed that RNF213 deficiency reduced the activation of critical regulators for IFN-I production IRF3 and TBK1 by using western blot experiments (Fig 2). In addition, we also found that over-expression of RNF213 results in increased expression of ISGs by using qRT-PCR（S2 Fig）.**

**Regarding the ubiquitination target of RNF213, we found that RNF213 deficiency did not affect the expression of ISGs during IFN-β stimulation (Fig 2) indicating that the target of RNF213 might be one of critical regulators for INF-I production (upstream of IFNAR, Fig 7). Following this rationale, we defined TRAF3 as the direct target of RNF213 by using multiple Co-IP and ubiquitination experiments (Fig 5A-5B, S7A Fig, Fig 6A). In the revised manuscript, we also confirmed the ubiquitination site K160 of TRAF3 mediated by RNF213 was essential for ISGs expression (Fig 6F-6G). Overall, our data provided multiple evidences that RNF213 directly ubiquitinated TRAF3 for promoting the expression of ISGs. We have added new data to Figure S2A, B; Figure 6F, G.**

3. The bone marrow transplantation experiments (Fig. 3J-K) require additional controls. The authors should include data demonstrating successful engraftment, chimerism quantification, and include flow cytometry data and analysis of the immune cell populations from chimeric mice.

**We thank the reviewer for this suggestion. We performed bone marrow transplantation** **(BMT) between WT** **(CD45.1^+^) and *Rnf213^–/–^* (CD45.2^+^) mice to examine the engraftment efficiency. Six weeks after transplantation, we performed flow cytometry analysis and confirmed that donor-derived bone marrow cells were predominantly existed in the bone marrow, blood, and spleen of recipient mice (S5B Fig). In addition, we performed western blot analysis of RNF213 in chimeric mice, and found that RNF213 was notably detected in the spleen from *Rnf213^–/–^* mice that received bone marrow from WT mice, but not detected in the spleen from WT mice that received bone marrow from *Rnf213^–/–^* mice (S5C Fig).**

**These data collectively indicate that the transplantation system is successful in our experiments. We have added these new data regarding transplantation controls to Figure S5B, C.**

**S5B Fig**

**S5C Fig**

4. The mechanism by which RNF213-mediated K27-linked ubiquitination of TRAF3 leads to increased type I interferon production is not fully elucidated. Additional experiments addressing the functional consequences of this modification on the activity or stability of TRAF3 are necessary.

**We thank the reviewer for this suggestion. We have designed additional experiments to address this question. We transfected WT and mutant TRAF3 (K156R, K160R) into *Traf3^–/–^* MSFs and evaluated ISGs and TJ-related proteins expression in response to RNF213 overexpression. We found that the expression of *Ifnb, Cxcl9, Cxcl10, Claudin-5, Occludin* and *Pecam1* was significantly higher in *Traf3^–/–^* MSFs co-transfected with WT TRAF3 or K156R mutant of TRAF3 and RNF213 than those in *Traf3^–/–^* MSFs co-transfected with K160R mutant of TRAF3 and RNF213 in response to *A. baumannii* infection. Consistently, the K160R mutation of TRAF3 impaired its ability to activate TBK1 and IRF3 compared to WT TRAF3 and K156R. We have added these new data to Fig 6F and 6G and S8 Fig.**

**Figure 6F**

**Figure 6G**

**S8 Fig**

5. Using macrophages derived from Rnf213 deficient mice to generalize the findings to human health is not convincing. Validation of this conclusion in a human cell line would better demonstrate the translatability of this data.

**We thank the reviewer for the suggestions. We have performed overexpression of RNF213in both mouse MSFs and human umbilical vein endothelial cells (HUVECs) to validate the key roles of RNF213 in regulating IFN-I signaling. We found that RNF213 overexpression in mouse MSFs and HUVECs enhanced *A. baumannii* infection-induced the expression of *Cxcl9, Cxcl10*, and *Irf1* but not *Tnfa* or *Il1a*.** **We have added these new data to S2A-2B Fig.**

**S2A Fig**

**S2B Fig**

**Minor Issues: Editorial and Data Presentation Modifications**

1. The Western blot in Figure 2D requires quantification across multiple experiments with appropriate statistical analysis.

**We have performed quantification of the Western blot bands from three independent experiments. We have added the quantification data and statistical analysis to S3C-S3D Fig and related figure legends.**

**S3C Fig**

**S3D Fig**

2. In Figure 4, the authors should include size markers on all immunofluorescence images and provide more detailed information about image acquisition and processing in the methods section.

**We have added more detailed information describing image acquisition and quantification analysis of confocal microscopy images in the methods section. We added size markers on all immunofluorescence images.**

**Figure 4G and 4J**

3. Lines 353-355: The sentence beginning with "In this study, we define RNF213..." is unclear.

**We have added more detailed information in the beginning of discussion section.**

4. The discussion section (starting line 352) would be strengthened by acknowledging the study limitations more directly and discussing other known regulators of the blood-brain barrier and how they may correlate to the provided mechanism.

**We thank the reviewer for this suggestion. As suggested, we have revised the Discussion to state the study's limitations and add sentence with references to better establish the relevance of our findings (line 389-400; line 435-449).**

5. The methods for primary BMEC isolation and culture, the criteria for quantifying BBB disruption, and details for how the RNA-seq was analyzed could be expounded upon.

**We thank the reviewer for these suggestions. We have added more information describing the methods for primary BMEC isolation and culture (line 508-521), the criteria for quantifying BBB disruption (line 538-549), and details for how the RNA-seq was analyzed in the methods section (line 665-678).**

6. Several figure legends lack sufficient detail about experimental conditions and numbers of replicates.

**We have carefully revised all figure legends throughout the manuscript, added** **numbers of replicates for each experiment and more detailed descriptions of the experimental conditions.**

7. Supplementary Table 1 should include adjusted p-values for the RNA-seq analysis.

**We thank the reviewer for this suggestion. We performed p-value analysis for the RNA-seq data and defined candidate DEGs. We also performed qRT-PCR analysis and verified the expression of *Rnf213*, one of the most significantly regulated genes by IFN-I signaling.**

8. Throughout the manuscript, the authors inconsistently use "A. baumannii" and "A.bau." This should be standardized.

**We have carefully checked and made consistence throughout the manuscript.**

9. For Figures 2B and 2C, error bars should be included for all time points.

**We thank the reviewer for this suggestion. We have now added error bars to all time points in Figures 2B and 2C.**

**Figure 2B and 2C**

10. The RT-qPCR data should include information about the housekeeping genes used and how they were validated for these experimental conditions.

**We thank the reviewer for the suggestion regarding the RT-qPCR data.** **We have now included the specific housekeeping genes used in our RT-qPCR experiments in the Methods and Legend sections.** **We selected HPRT (hypoxanthine guanine phosphoribosyltransferase) as our reference gene based on its well-documented stability as a housekeeping gene, ensuring reliable normalization of target gene expression across all experimental groups.**

11. Statistical analyses should be more clearly described in the methods section, including specific tests used for each type of comparison.

**We sincerely thank the reviewer for your suggestion regarding the description of statistical analyses in the Methods section. We have revised the Methods section and legends to explicitly describe the specific statistical tests used for each type of comparison in our study.**

Reviewer #2:

**Summary**

In this study the authors show how RNF213 deficiency impairs type I IFN production and decreases expression of ISGs in response to Acinetobacter infection. They reveal that RNF213 is required for efficient TBK1 and IRF3 phosphorylation upon infection with Acinetobacter or stimulation with LPS and further reveal that RNF213 interacts with TRAF3, but not associated signaling molecules TRIF, TBK1 or IRF3.

Analysis of TRAF3 ubiquitylation suggests that RNF213 mediates ubiquitylation at K160.

RNF213 is required to restrict the burden of Acinetobacter in the brain of infected mice (but not the spleen) and this is associated with a loss of blood brain barrier in the RNF213 KO.

The data is well-presented and by and large well controlled. The findings are of interest given the recent discovery that RNF213 is an immune sensor that controls numerous and diverse pathogens. The findings are significant.

**We thank the reviewer found our study interesting and significant.**

**Major Issues: Key Experiments Required for Acceptance**

The main gap regarding the model and study conclusions is that figure 5 lays out that RNF213 and TRAF3 interact with each other, and that TRAF3 is ubiquitylated at K160, yet whether mutation of K160, so that it can’t be ubiquitylated, impacts downstream phenotypes including TBK1/IRF3 phosphorylation upon Acinetobacter infection, IFN/ISG production and expression of tight junction proteins is not analysed. This is important, as TRAF3 KO’s are not analysed either.

**We thank the reviewer for this valuable suggestion. We have designed additional experiments to address this question. We transfected WT and mutant TRAF3 (K156R, K160R) into *Traf3^–/–^* MSFs and evaluated ISGs and TJ-related proteins expression in response to RNF213 overexpression. We found that the expression of *Ifnb, Cxcl9, Cxcl10, Claudin-5, Occludin* and *Pecam1* was significantly higher in *Traf3^–/–^* MSFs co-transfected with WT TRAF3 or K156R mutant of TRAF3 and RNF213 than those in *Traf3^–/–^* MSFs co-transfected with K160R mutant of TRAF3 and RNF213 in response to *A. baumannii* infection. Consistently, the K160R mutation of TRAF3 impaired its ability to activate TBK1 and IRF3 compared to WT TRAF3 and K156R. We have added these new data to Fig 6F and 6G and S8 Fig.**

**Figure 6F**

**Figure 6G**

**S8 Fig**

Related to this, whilst an RNF213 mutant is tested in the ubiquitylation of TRAF3 assay, unless a TRAF3 catalytic mutant is also analysed, does it not remain possible that TRAF3 mediates its own ubiquitylation, perhaps upon interaction or modification by RNF213?

Then, given sentences such as this “Our findings elucidate the potential functions of the RNF213–IFN-I–BBB axis in moyamoya disease development.” I would have expected the analysis of RNF213 mutations associated in moyamoya disease to have been analysed for TRAF3 ubiquitylation, IFN signalling and tight junction formation.
**We thank the reviewer for raising this question.** **We agree with you that TRAF3 might be ubiquitinated by itself or other E3 ligases in addition to RNF213. To examine the RNF213 mutations associated in moyamoya disease for TRAF3 ubiquitination, we chose the mouse homologous site (W3974) with the amino acid W4024 within the human RNF213 RING domain, which is found to be mutated in MMD patients and essential for E3 ligase activity [1] (S7D Fig). Our data revealed that W3974R mutant impairs the K27-linked ubiquitination of TRAF3, and the K27-linked ubiquitination of TRAF3 is important for TRAF3 activation and downstream ISGs expression induced by RNF213 overexpression (Fig. 6C-G).**

**Furthermore, the homo- or hetero-dimer RING domain-interaction confers E3 ubiquitin ligase activity [2]. TRAF3 contains an N-terminal RING domain, and the RING domain contains seven conserved cysteines and one histidine which are involved in the coordination of two atoms of zinc. Mutation of the last residue of the domain compromises the stability of the adjacent cysteine, affecting the coordination of the zinc atom [3-5] (S7B Fig). We generated TRAF3 Q91A mutant which is a functional linchpin within RING domain (S7C Fig), and found that the Q91A mutation blocked the interaction between RNF213 and TRAF3 (Fig 5F).**

**Due to the lack of information regarding K27-linked polyubiquitination of TRAF3 by itself, we could not rule out the possibility that the contributing roles of TRAF3 catalytic activity. We have revised our description to“RNF213 potentially mediates BBB in moyamoya disease development by targeting TRAF3 for the regulation of IFN-I signaling”.**

**S7B Fig**

**S7C Fig**

**S7D Fig**

**Figure 5F**

**Minor Issues: Editorial and Data Presentation Modifications**

Figure 5A – the IP of RNF213 by TRAF3 shows some RNF213 coming down without any TRAF3, implying a degree of stickiness. Ideally a different tagged protein would be used for the “bait” in the IP assay in both this figure and 5B.

**We have repeated the experiment and replaced Figure 5A with the new data.**

**Figure 5A**

Figure 5C – not hugely convinced on the interaction becoming enhanced with infection. If RNF213 and TRAF3 interact in steady state, what is causing the induction of the signal upon infection/LPS?

**We thank the reviewer for this question. RNF213 expression is upregulated in cells infected with *A. baumannii*. This increased expression may provide a plausible explanation for the enhanced co-immunoprecipitation signal induced by** ***A. baumannii* infection. We have revised the description in the results.**

Figure 5D - Microscopy appears to show the protein everywhere in the cell, perhaps as it is heavily overexpressed. From this condition, then, it doesn’t seem possible to discern whether the proteins are really colocalising and as one can’t tell which cells are infected, whether this is something induced by infection.

**We thank the reviewer for this suggestion. We performed new experiments by reducing the expression level of transfected plasmids and confirmed the colocalization of RNF213 with TRAF3. We have replaced Figure 5D with the new data.**

**Figure 5D**

The text should make clear which catalytic mutant of RNF213 is used in figure 6 for analysis of TRAF3 ubiquitylation. The figure legend states it is W3974R but it should be made clear in the text if this is the RING domain or the non-canonical RZ finger in the E3 shell. The fact it has two domains that mediate ligase activity should also be mentioned in the introduction and discussion. This is important regarding the claim that TRAF3 is the first host substrate of RNF213.

**We thank the reviewer for this suggestion. We have added more information in the introduction to the revised manuscript to describe how the RNF213 mutation sites were selected. Evidences suggest that multiple mutations in the RNF213 RING domain of MMD patients have been reported. Interestingly, the amino acid W4024 is exposed at the surface of the RING domain (the homologous site in mice is W3974), and located within the central helix that connects the two zinc coordination sites. Since the coordination of the two atoms of zinc by the RING domain is crucial for E3 ligase activity, mutations in this residue impair the RING-E2 interaction necessary for E3 ubiquitin ligase activity [1, 3, 6].**

Reviewer #3:

**Summary**

Moyamoya disease is a cerebrovascular disease that can require surgery and be fatal. RNF213 is a major susceptibility gene, yet disease penetrance is extremely low. This suggests an additional, potentially environmental, factor contributes to the disease. The recently established role of RNF213 in microbial resistance implies pathogen infection might be connected, but a basis for this is unknown. The findings in this study suggest RNF213 plays a key role in BBB integrity during infection, providing a potential link to the aetiology of Moyamoya. This is an important finding. Moyamoya patient Mutations are found throughout the protein but cluster in the RING domain. However, the RING domain lacks E3 activity as it can be deleted with no defect in autoubiquitination. It also lacks a key linchpin residue required for RING E3 activity. Hence, the function of the RING domain, and its role in Moyamoya, remain unclear. The finding that the RING domain protein TRAF3 interacts with RNF213 and becomes ubiquitinated might explain the function of the RNF213 RING domain, but further experimentation is required (see below).

Overall, the paper is poorly written and lacks important citations throughout the results section. Without these its not clear what findings are novel. Indeed, some are claimed as such when they are not e.g. it is already known that RNF213 is an ISG. Figure legends are insufficient to appreciate what experiment is being performed and the methods section is not comprehensive enough to allow reproduction of the experiments.

Some aspects of the paper are very strong and convincing, but some require extensive revision or should be omitted entirely from the paper (RNA seq data).

**We thank the reviewer found our study important. We sincerely appreciate the reviewer's comprehensive critique and valuable suggestions to our manuscript. We have extensively revised our manuscript in writing, citation, legends, methods, and etc.** **Please check our responses to all the specific aspects below.**

**Major Issues: Key Experiments Required for Acceptance**

Initially, RNA seq data showing genes upregulated in WT vs IFNAR KO cells are related to innate immune responses, which is to be expected so there is nothing new presented here. There are also multiple issues with the presentation of the RNA-seq data.
• Media (Fig. S1A) and 0 hour treatment (Fig. S1B) are referred to and the numbers of upregulated proteins are different for both analyses. Are these different datasets? This is not explained properly.

**We thank the reviewer pointing out the inconsistent labeling. We have revised the labeling for Fig. S1A and Fig. S1B. The venn diagram in Fig. S1B exhibits the enrichment of differently expressed genes in Fig. S1A.**

• Figure 1A heatmap is incomprehensible. What is the reference for the relative fold changes (x/y)? Is y a reference protein? is it an earlier time point?? This lack of precision is unacceptable. Presumably the fold change is on a log2 scale but this is not specified. The colour ramp (red or blue) is also inappropriate to reveal changes the authors might be referring to.

**We thank the reviewer for raising these questions. We have revised the gene list in Figure 1A and the heatmap presentation with more detailed labeling. In the 93 commonly upregulated genes in WT versus *Ifnar^–/–^* BMDMs under both uninfected and *A. baumannii*-infected conditions, *Rnf213* is one of the most highly expressed and significantly regulated genes by IFNAR signaling. The heatmap in Figure 1A exhibited the top 40 genes highly expressed in WT BMDMs and significantly regulated by IFNAR signaling in both uninfected and *A. baumannii*-infected conditions (S1C Fig).**

**The expression values of each gene were standardized (z-score normalization) across samples to facilitate comparison. The color scale represents relative expression levels, where blue indicates lower expression, white represents median expression, and red indicates higher expression.**

**Figure 1A**

**S1C Fig**

Figure 1B RT-PCR data shows time dependent increase in RNF213 expression for a range of infection models and is strongly dependent on IFNAR. Legend should state what *Hprt* is. I am assuming this is a housekeeping protein used as a reference. Western blot data nicely validates the RT-PCR data. However, this is not surprising considering RNF213 is a known ISG. This should be stated along with an appropriate reference.
**We thank the reviewer for this suggestion. We have added information for *Hprt* (Hypoxanthine** **guanine phosphoribosyltransferase) in legend and method sections. We also added references to describe that RNF213 as an ISG which is induced by pathogen infection and stimuli [7, 8].**

siRNA experiments then show that RNF213 is required for interferon signalling by measuring a decrease in the markers Cxcl9, Cxcl10 and Irf1. It should be stated what these markers represent, and a reference is once again required. The inflammatory cytokines Tnfa and II1a were unaffected by RNF213 KD so it is asserted that RNF213 mediates its effects upstream of IFNAR signalling.

**We thank the reviewer for this suggestion. We have added the information and references to chemokine genes *Cxcl9, Cxcl10*, and transcription factor *Irf1*, which are generally used responsive genes upon IFNAR signaling activation [9, 10].**

S2 legends are once again inadequate and should state that the x axis corresponds to the time following infection, if that is what it is. Statistical tests used and P value assigned to *** should also be stated.

**We thank the reviewer for pointing out these issues. We have added more detailed information to state that the x axis corresponds to the time following *A. baumannii* (50 MOI) infection in S2 Fig legends. We have also revised the legends to explicitly describe the specific statistical tests used in our study.**

Due to the link between RNF213 with Moyamoya disease, bacterial load in the brain is measured in WT and RNF213 KO mice and is found elevated in the brain but not in the spleen, suggestive of organ-specificity. Bone marrow cross transplantation experiments are next carried out. This experiment needs to be more clearly explained. The below is also very difficult to follow:
“Interestingly, WT mice that received bone marrow from either WT or Rnf213–/– mice exhibited a significantly lower bacterial load in the brain than did Rnf213−/− mice that received bone marrow from either WT or Rnf213–/– mice (Fig 3J), whereas the bone marrow cells isolated from WT and Rnf213–/– mice had a comparable capacity to regulate the bacterial burden in the brain in WT and Rnf213–/– mice (Fig 3J)”.
This experiment lacks important controls demonstrating bone marrow has been completely destroyed as the findings could be explained by residual marrow in WT samples following radiation treatment. This caveat should be clearly stated.

**We thank the reviewer for this suggestion. We performed bone marrow transplantation (BMT) between WT (CD45.1^+^) and *Rnf213^–/–^* (CD45.2^+^) mice to examine the engraftment efficiency. Six weeks after transplantation, we performed flow cytometry analysis and confirmed that donor-derived bone marrow cells were predominantly existed in the bone marrow, blood, and spleen of recipient mice (S5B Fig). In addition, we performed western blot analysis of RNF213 in chimeric mice, and found that RNF213 was notably detected in the spleen from *Rnf213^–/–^* mice that received bone marrow from WT mice, but not detected in the spleen from WT mice that received bone marrow from *Rnf213^–/–^* mice (S5C Fig).**

**These data collectively indicate that the transplantation system is successful in our experiments. We have added these new data regarding transplantation controls to Figure S5B, C.**

**S5B Fig**

**S5C Fig**

Strikingly, a compromised blood brain barrier is seen in RNF213 KO samples, as inferred from leakage of Evans blue dye into the organ. Quantitative confocal microscopy shows that baumanni infection enhances EBD leakage and this is exacerbated in RNF213 KO samples.

To test the hypothesis that brain leakage is due to RNF213 having a role in regulating TJ-related proteins, their levels (Claudin-5, Pecam1, Occludin) are measured by RT-PCR in response to infection. Analysis shows that baumannii infection upregulates TJ markers and this is suppressed in RNF213 KO cells. It is not clear if upregulation of TJ proteins upon infection of WT cells is statistically significant. P values should be assigned to the plots.

**We thank the reviewer for this suggestion. We have added statistical analysis for WT cells between control and infection, which were statistically significant (*P<0.001*) for *Rnf213,*** ***Claudin-5, Pecam1*, and *Occludin* (Figure 3G, 4C, 4I).**

**Figure 3G**

**Figure 4C**

**Figure 4I**

The microscopy does not show a clear increase in Claudin-5 in WT vs WT infected samples. The significance of this should also be calculated. Couldn’t the microscopy experiment be repeated with Occludin, which is stimulated more robustly upon infection?

**We appreciate the reviewer's careful evaluation of our microscopy data and the suggestions for improvement. We added the quantification analysis of anti-Claudin-5 immunofluorescence data to Figure 3I, 4K.**

**Figure 3I**

**Figure 4K**

**In addition, we performed anti-Occludin confocal microscopy analysis. We have added the representative images and quantification data to S4A-S4B Fig and S6A-S6B Fig.**

**S4A-S4B Fig**

**S6A-S6B Fig**

Compromised expression of TJ markers upon infection is also observed in brains from RNF213 KO animals.

Similar brain phenotypes are observed with IFNAR KO mice. However, the statement that the findings “indicate that RNF213 modulates blood-brain barrier integrity via IFN-I signalling” is overstated and should be toned down.
**We thank the reviewer for this suggestion. We have revised the sentence to “RNF213 is essential for blood-brain barrier integrity upon *A. baumannii* infection via targeting TRAF3 and regulation of IFN-I signaling”.**

Section RNF213 targets TRAF3
Where are the data showing RNF213 deficiency inhibits TBK1 phosphorylation? This is an essential piece of data. Following from this, co-IPs in HEK cells with a panel proteins involved in IFN production are carried out and TRAF3 is found to interact. Truncations clearly show that the N-terminal 109 residues of TRAF3 containing the RING is sufficient for interaction. The co-IP is also observed with endogenous proteins. Although for this experiment a control demonstrating that the TRAF3 antibody doesn’t cross react with RNF213 is missing. RNF213 levels are higher in Abaumanii infected cells so this might also explain why the apparent co-IP is higher. Microscopy data do support colocalization in cells.

**We thank the reviewer for the suggestions. We have the data showing RNF213 deficiency reduces TBK1 phosphorylation upon infection and stimulation in both MSFs and BMECs in Figure 2D-2E and S3C-S3D Fig.**

**Figure 2D-2E**

**S3C-S3D Fig**

**We also performed immunoprecipitation with an anti-TRAF3 antibody to analyze the interaction of endogenous RNF213 with TRAF3 in WT and *Rnf213^–/–^* MSFs with *A. bauman*nii infection. We have the control experiment to confirm that TRAF3 antibody doesn’t cross react with RNF213, please check the data below.**

It is next investigated whether RNF213 ubiquitinates TRAF3. Apparent ubiquitination of TRAF3 is observed and by using a panel of single lysine HA-Ub mutants, the data suggest K27-linkages predominate. A reverse experiment with a K27R mutant substantially reduces ubiquitination, further supporting modification with K27 linkages. To determine if RNF213 ligase activity is responsible a “catalytic site mutant (M)” is tested. There is no reference to the origin of this mutant, but the legend states it is a W3974R mutation. This is located in the RING domain. The authors must state how this mutation was chosen. Moreover, the RING domain does not contribute to RNF213 activity which depends on its RZ domain where it uses a non-RING transthiolating mechanism (Ahel et al. Biorxiv). The RING domain also lacks a hydrophilic linchpin residue required for RING E3 activity. Thus, the function of the RING domain remains unclear but is clearly relevant to Moyamoya disease because disease mutations cluster within it.

**We thank the reviewer for this suggestion and constructive comments. We have added more information in the introduction to describe how the RNF213 mutation sites were selected. Evidences suggest that multiple mutations in the RNF213 RING domain of MMD patients have been reported. Interestingly, the amino acid W4024 is exposed at the surface of the RING domain (the homologous site in mice is W3974), and located within the central helix that connects the two zinc coordination sites. Since the coordination of the two atoms of zinc by the RING domain is crucial for E3 ligase activity, mutations in this residue impair the RING-E2 interaction necessary for E3 ubiquitin ligase activity [1, 3, 6].**

**S7D Fig**

Whether TRAF3 in isolation is an active E3 is also unclear. RING domains often require homo or heterodimerisation (e.g. BRCA1-BARD1) to become active. An exciting possibility is that to become active, the RNF213 RING domain heterodimerises with the TRAF3 RING domain, which does have a functional Gln linchpin (human Gln92). The authors should test if Gln92, or the equivalent tryptophan mutation in TRAF3 is required, in addition to RNF213, for TRAF3 ubiquitination.？

**We thank the reviewer for this suggestion. Our data revealed that W3974R mutant impairs the K27-linked ubiquitination of TRAF3, and the K27-linked ubiquitination of TRAF3 is important for TRAF3 activation and downstream ISGs expression induced by RNF213 overexpression (Fig. 6C-G). Furthermore, the homo- or hetero-dimer RING domain-interaction confers E3 ubiquitin ligase activity [2]. TRAF3 contains an N-terminal RING domain, and the RING domain contains seven conserved cysteines and one histidine which are involved in the coordination of two atoms of zinc. Mutation of the last residue of the domain compromises the stability of the adjacent cysteine, affecting the coordination of the zinc atom [3-5] (S7B Fig). We generated TRAF3 Q91A mutant which is a functional linchpin within RING domain (S7C Fig), and found that the Q91A mutation blocked the interaction between RNF213 and TRAF3 (Fig 5F).**

**S7B Fig**

**S7C Fig**

**Figure 5F**

**Minor Issues: Editorial and Data Presentation Modifications**

1.	Guey S, Kraemer M, Hervé D, Ludwig T, Kossorotoff M, Bergametti F, et al. Rare RNF213 variants in the C-terminal region encompassing the RING-finger domain are associated with moyamoya angiopathy in Caucasians. European Journal of Human Genetics. 2017;25(8):995-1003. doi: 10.1038/ejhg.2017.92.

2.	Hu Q, Botuyan MV, Zhao D, Cui G, Mer E, Mer G. Mechanisms of BRCA1–BARD1 nucleosome recognition and ubiquitylation. Nature. 2021;596(7872):438-43. doi: 10.1038/s41586-021-03716-8.

3.	Garcia-Barcena C, Osinalde N, Ramirez J, Mayor U. How to Inactivate Human Ubiquitin E3 Ligases by Mutation. Frontiers in Cell and Developmental Biology. 2020;8. doi: 10.3389/fcell.2020.00039.

4.	Deshaies RJ, Joazeiro CA. RING domain E3 ubiquitin ligases. Annual review of biochemistry. 2009;78:399-434. Epub 2009/06/06. doi: 10.1146/annurev.biochem.78.101807.093809. PubMed PMID: 19489725.

5.	Ni C-Z, Oganesyan G, Welsh K, Zhu X, Reed JC, Satterthwait AC, et al. Key Molecular Contacts Promote Recognition of the BAFF Receptor by TNF Receptor-Associated Factor 3: Implications for Intracellular Signaling Regulation. The Journal of Immunology. 2004;173(12):7394-400. doi: 10.4049/jimmunol.173.12.7394.

6.	Takeda M, Tezuka T, Kim M, Choi J, Oichi Y, Kobayashi H, et al. Moyamoya disease patient mutations in the RING domain of RNF213 reduce its ubiquitin ligase activity and enhance NFκB activation and apoptosis in an AAA+ domain-dependent manner. Biochemical and biophysical research communications. 2020;525(3):668-74. doi: 10.1016/j.bbrc.2020.02.024.

7.	Costa B, Becker J, Krammer T, Mulenge F, Durán V, Pavlou A, et al. Human cytomegalovirus exploits STING signaling and counteracts IFN/ISG induction to facilitate infection of dendritic cells. Nature communications. 2024;15(1). doi: 10.1038/s41467-024-45614-3.

8.	Yang X, Zhu X, Sheng J, Fu Y, Nie D, You X, et al. RNF213 promotes Treg cell differentiation by facilitating K63-linked ubiquitination and nuclear translocation of FOXO1. Nature communications. 2024;15(1). doi: 10.1038/s41467-024-50392-z.

9.	Moledina DG, Obeid W, Smith RN, Rosales I, Sise ME, Moeckel G, et al. Identification and validation of urinary CXCL9 as a biomarker for diagnosis of acute interstitial nephritis. J Clin Invest. 2023;133(13). Epub 2023/07/03. doi: 10.1172/jci168950. PubMed PMID: 37395276; PubMed Central PMCID: PMCPMC10313360.

10.	Koper OM, Kamińska J, Sawicki K, Kemona H. CXCL9, CXCL10, CXCL11, and their receptor (CXCR3) in neuroinflammation and neurodegeneration. Advances in clinical and experimental medicine : official organ Wroclaw Medical University. 2018;27(6):849-56. Epub 2018/06/13. doi: 10.17219/acem/68846. PubMed PMID: 29893515.
