# Supplementary material for: RNF213 regulates blood‒brain barrier integrity by targeting TRAF3 for type I interferon activation during A. baumannii infection
Source: PLoS Pathog. 2025 Jul 7;21(7):e1013333. doi: 10.1371/journal.ppat.1013333 (PMC12251097; doi:10.1371/journal.ppat.1013333)
Supplement: Attachment — Submitted filename: Response to Reviewers_250617.doc [file ppat.1013333.s012.doc]

Your revised manuscript has now been seen by the three original reviewers. All acknowledge the substantial efforts you havemade to address their initial comments.

However, two reviewers have remaining concerns that must be addressed before the manuscript can be accepted for publication. Many of these points can likely be resolved through a more detailed discussion in the manuscript. That said, I would like to emphasize that Reviewer 2’s concerns regarding the potential lack of specificity of a key reagent in your study—the TRAF3 antibody—will require convincing experimental evidence to be fully resolved.

1. **We performed immunoprecipitation with an anti-IgG antibody as control to analyze the interaction of endogenous RNF213 with TRAF3 in WT and *Rnf213^–/–^* MSFs in response to *A. bauman*nii infection. We have the control experiment to confirm that TRAF3 antibody is specifically bringing down RNF213.**

**Please check our responses to all the specific aspects below:**

Reviewer #2:

**Summary**

The authors have added a substantial amount of data to the manuscript addressing many of the major concerns.

**Major Issues: Key Experiments Required for Acceptance**

I have only one remaining query related to figure 5C - reviewer comments noted that to demonstrate a well controlled IP of RNF213 with TRAF3 requires further controls, e.g. to demonstrate that TRAF3 antibody isn't non-specifically bringing down RNF213 in the experiment - the provided blot using a TRAF3 antibody on input samples does not seem to address this and it is perhaps important given that a band of the molecular weight of RNF213 is present in the RNF213 KO IP lanes.

**We thank the reviewer for raising this question.** **We repeated immunoprecipitation with an anti-IgG antibody as a negative control to analyze the interaction of endogenous RNF213 with TRAF3 in WT and *Rnf213^–/–^* MSFs in response to *A. bauman*nii infection. We have the control experiment to confirm that TRAF3 antibody is specifically bringing down RNF213, please check the data below.**

**Figure 5C**

**Minor Issues: Editorial and Data Presentation Modifications**

I would like to see further discussion regarding TRAF3 RING mutant Q91A and lack of interaction to RNF213 -does it imply TRAF3 ligase activity is required for stable interaction? I also find this sentence vague "In this study, we show that the interaction between RNF213 and TRAF3 through RING domain may ignite the E3 ubiquitin ligase activity [51], and RNF213 ..." are the authors suggesting that the RING heterodimerisation between RNF213 and TRAF3 might create a catalytically active RING domain and if so, this should be stated more clearly.

**We thank the reviewer for this suggestion. We have added a discussion of these possibilities in the revised manuscript (line 383-387, 477-481).**

**“We tested whether RNF213–TRAF3 heterodimerization might create a catalytically active RING domain by mutating the Q91 linchpin residue. However, the Q91A mutation abolished RNF213–TRAF3 interaction, suggesting that heterodimerization might be required for TRAF3 ubiquitination, which needs further investigation.”**

**“In this study, we demonstrate that the RING domain-mediated interaction between RNF213 and TRAF3 activates E3 ubiquitin ligase activity, suggesting that the RING heterodimerization between RNF213 and TRAF3 might create a catalytically active RING domain.”**

Reviewer #3:

**Summary**

The authors have made satisfactory efforts to improve the paper. Some elements remain overstated and lack objective discussion.

**Major Issues: Key Experiments Required for Acceptance**

The following should be considered before acceptance:

Figure 1A – Please label the color ramp with “normalized Z-score” for clarity.

**We thank the reviewer for this suggestion. We have marked the color gradient with the normalized Z-score and added corresponding explanations in the Figure legend (line 721-723).**

**Figure 1A**

Results – IFN-I regulation of Rnf213:

The sentence:

"Taken together, these results indicate that IFN-I mediates the increase in Rnf213 expression induced by pathogen infection and stimuli，which is consistent with previous study that RNF213 as one of ISGs [29, 30]."

could be more clearly phrased. Suggested revision:

“Taken together, these results indicate that type I interferon (IFN-I) mediates the pathogen-induced upregulation of Rnf213, consistent with previous studies identifying Rnf213 as an interferon-stimulated gene (ISG) [29, 30].”

**We thank the reviewer for this suggestion and have changed the sentence to “Taken together, these results indicate that IFN-I mediates the pathogen-induced upregulation of *Rnf213*, consistent with previous studies identifying *Rnf213* as an interferon-stimulated gene (ISG) [29, 30]” in the results section (line 176-179).**

Results – TBK1 Phosphorylation:

Thank you for highlighting the TBK1 phosphorylation data. To improve clarity for the reader, please reference these data again in the “RNF213 Targets TRAF3” section. i.e.:

“RNF213 deficiency inhibited TBK1 phosphorylation and IFN-I production in response to LPS stimulation and A. baumannii infection, but not in response to IFN-β treatment, suggesting that RNF213 positively regulates IFN-I signaling by acting on targets upstream of IFNAR (Fig 2D and 2E, S3C and S3D).”

**We thank the reviewer for this constructive suggestion. To improve clarity, we have now referenced the TBK1 phosphorylation data in the "RNF213 Targets TRAF3" section (line 324-327).**

RNF213 Interaction with TRAF3:

It should be mentioned that the observed increase in RNF213–TRAF3 interaction following *A. baumannii* infection may be, at least in part, due to elevated RNF213 expression levels.

**We have added the suggested clarification regarding RNF213 expression levels in the revised manuscript (line 336-340) as follows:**

**“****Notably, our results revealed that RNF213 interacted with TRAF3 in control and *A. baumannii*-infected WT MSFs, and increased RNF213–TRAF3 interaction following *A. baumannii* infection might be partially due to elevated RNF213 expression levels (Figure 5C).**

RING Dimerization and E3 Activity:

The statement:

“Furthermore, the homo- or hetero-dimer RING domain-interaction confers E3 ubiquitin ligase activity [2].” is unclear.

Hu et al. (Ref 2) do not demonstrate that homodimerization of BRCA1 or BARD1 results in functional E3 activity. Therefore, the possibility that RNF213 requires heterodimerization for E3 activity remains valid.

The authors should state that they tested whether RNF213–TRAF3 dimerization is required for E3 ligase activity by mutating the Q91 linchpin residue. This discussion would be more appropriate in the “RNF213 regulates the K27-linked polyubiquitination of TRAF3” section.

Given that the Q91 mutation abolished interaction, it is unfortunate this prevented formal testing. Nonetheless, it should be clearly stated that heterodimerization may still be required for TRAF3 ubiquitination, and the discussion should reflect this possibility more objectively.

**We thank the reviewer for this suggestion and constructive comments. We have added the suggested discussion regarding RING dimerization and E3 activity in the revised manuscript (line 383-387) as follows:**

**“We tested whether RNF213–TRAF3 heterodimerization might create a catalytically active RING domain by mutating the Q91 linchpin residue** **(Fig 5F). However, the Q91A mutation abolished RNF213–TRAF3 interaction, suggesting that heterodimerization might be required for TRAF3 ubiquitination, which needs further investigation.”**

W3974R Mutation and E3 Activity:

The manuscript still asserts that W4024 (W3974 in human RNF213) is essential for E3 oligase activity. However, this conclusion may be overstated.

• Guey et al. (Ref 1) speculate that the W4024R mutation impairs activity based on analogy to other RING E3s but do not directly test RNF213 activity.

• Garcia-Barcena et al. is a review article and does not discuss RNF213 specifically.

• Takeda et al. (Ref 3) assess the W4024R variant using a GST-RING fusion, which may artificially enhance activity due to GST dimerization. Although they observe modest activation of Ubc13/Uev1a, the physiological relevance is unclear. Moreover, NF-κB activation was seen even with E3-dead mutants, including W4024R.

Thus, it should be stated that W3974R is predicted to be essential for E3 activity, which is what Ref 26 (used to support the claim) asserts.

**We have added the suggested clarification regarding W3974R mutation and E3 activity in the revised manuscript (line 376-378) as follows:**

**“Notably, the W3974R mutation in RNF213 abolished TRAF3 polyubiquitination, indicating that W3974 plays important roles for E3 ubiquitin ligase activity (Fig 6C).”**
